# Brain serotonin 1A receptor binding: relationship to peripheral blood DNA methylation, recent life stress and childhood adversity in unmedicated major depression

**DOI:** 10.1192/bjp.2023.13

**Published:** 2023-09

**Authors:** Hanga Galfalvy, Eileen Shea, Jacqueline de Vegvar, Spiro Pantazatos, Yung-yu Huang, Ainsley K. Burke, M. Elizabeth Sublette, Maria A. Oquendo, Francesca Zanderigo, Jeffrey M. Miller, J. John Mann

**Affiliations:** Department of Psychiatry, Columbia University Vagelos College of Physicians and Surgeons, New York, New York, USA; and Department of Biostatistics, Columbia University Mailman School of Public Health, New York, New York, USA; Upward Farms, New York, New York, USA; Department of Psychiatry, Columbia University Vagelos College of Physicians and Surgeons, New York, New York, USA; Department of Psychiatry, Columbia University Vagelos College of Physicians and Surgeons, New York, New York, USA; and Molecular Imaging and Neuropathology Area, New York State Psychiatric Institute, New York, New York, USA; Psychiatry Department, University of Pennsylvania, Perelman School of Medicine, Philadelphia, Pennsylvania, USA; Department of Psychiatry, Columbia University Vagelos College of Physicians and Surgeons, New York, New York, USA; Molecular Imaging and Neuropathology Area, New York State Psychiatric Institute, New York, New York, USA; and Department of Radiology, Columbia University, New York, New York, USA

**Keywords:** Serotonin 1A receptor, stress, childhood adversity, methylation, PET

## Abstract

**Background:**

Childhood and lifetime adversity may reduce brain serotonergic (5-HT) neurotransmission by epigenetic mechanisms.

**Aims:**

We tested the relationships of childhood adversity and recent stress to serotonin 1A (5-HT_1A_) receptor genotype, DNA methylation of this gene in peripheral blood monocytes and *in vivo* 5-HT_1A_ receptor binding potential (BP_F_) determined by positron emission tomography (PET) in 13 *a priori* brain regions, in participants with major depressive disorder (MDD) and healthy volunteers (controls).

**Method:**

Medication-free participants with MDD (*n* = 192: 110 female, 81 male, 1 other) and controls (*n* = 88: 48 female, 40 male) were interviewed about childhood adversity and recent stressors and genotyped for rs6295. DNA methylation was assayed at three upstream promoter sites (−1019, −1007, −681) of the 5-HT_1A_ receptor gene. A subgroup (*n* = 119) had regional brain 5-HT_1A_ receptor BP_F_ quantified by PET. Multi-predictor models were used to test associations between diagnosis, recent stress, childhood adversity, genotype, methylation and BP_F_.

**Results:**

Recent stress correlated positively with blood monocyte methylation at the −681 CpG site, adjusted for diagnosis, and had positive and region-specific correlations with 5-HT_1A_ BP_F_ in participants with MDD, but not in controls. In participants with MDD, but not in controls, methylation at the −1007 CpG site had positive and region-specific correlations with binding potential. Childhood adversity was not associated with methylation or BP_F_ in participants with MDD.

**Conclusions:**

These findings support a model in which recent stress increases 5-HT_1A_ receptor binding, via methylation of promoter sites, thus affecting MDD psychopathology.

Childhood adverse experiences, including abuse and neglect, increase risk for major depressive disorder (MDD) and suicidal behaviour in adulthood.^1^ Childhood adversity reduces brain serotonergic (5-HT) neurotransmission implicated in MDD, aggressive behaviour and suicidal behaviour.^2^ However, it is not clear how childhood adversity or recent life stressors may affect the serotonergic system via epigenetics or how epigenetic effects may increase the risk for MDD and other psychopathology in adulthood. Genetic polymorphisms of the 5-HT_1A_ receptor gene have been associated with MDD in a meta-analysis,^3^ and early-life stress may moderate this association,^4^ potentially via epigenetic effects, as it has been associated with DNA methylation of serotonergic pathway genes.^5^ One of the single nucleotide polymorphisms (SNPs) of the 5-HT_1A_ receptor gene (−1019C/G; rs6295)^6^ that regulate its expression^7^ has a G allele with lower affinity for inhibitory transcription factors.^8^ MDD is associated with a higher frequency of the rs6295 G/G genotype,^7^ and this genotype has been associated with greater 5-HT_1A_ binding in the raphe nuclei.^9^ Methylation of two CpG sites (−681 and −1007) increases autoreceptor expression, reducing serotonin release and affecting psychiatric illness and treatment response.^10^

We hypothesised a potential mediator role for DNA methylation at the regulatory sites listed above in the upstream promoter region of this gene (−681, −1007 and −1019) for the effect of childhood and recent stress on expression in the brain, potentially moderated by diagnosis for recent stress. To our knowledge, this is the first study to examine associations between (a) 5-HT_1A_ promoter genotype, childhood and recent life stress and (b) blood mononuclear cell DNA methylation and 5-HT_1A_ binding potential in brain, in order to better understand the role of 5-HT_1A_ binding as it relates to effects of stress on MDD.

## Method

### Participants

The sample included 192 participants with MDD (165 in a current major depressive episode) and 88 healthy volunteers (controls). MDD diagnosis was determined by DSM-IV criteria according to the Structured Clinical Interview for Axis I Disorders.^11^ Positron emission tomography (PET) 5-HT_1A_ receptor binding potential (BP_F_) was available for 119 participants (62%), including 69 with MDD and 50 controls. Subsets of the PET imaging data have been previously utilised in papers examining 5-HT_1A_ binding in people with depression and controls during an episode of MDD or between episodes.^12–15^ Exclusion criteria included fluoxetine use within 6 weeks of PET scanning, or exposure to a 5-HT_1A_ receptor agonist such as antipsychotic medications within 6 months of scanning. Participants in the MDD group on other antidepressant treatment at study enrolment underwent a medication washout and were drug-free for at least 2 weeks prior to neuroimaging, with the exception of short-acting benzodiazepines, which could be used as needed for anxiety/insomnia up to 72 h prior to scanning. The control participants had no history of DSM-IV Axis I or Axis II psychiatric disorders, no psychotropic medication exposure and no family history of a mood disorder. Exclusion criteria common to both groups included presence of significant active medical conditions, recent alcohol or other substance use disorder, dementia, neurological disease, head injury with loss of consciousness, pregnancy, first-degree family history of schizophrenia if younger than 33 years old and greater than three lifetime exposures to 3,4-methylenedioxymethamphetamine.

The authors assert that all procedures contributing to this work comply with the ethical standards of the relevant national and institutional committees on human experimentation and with the Helsinki Declaration of 1975, as revised in 2008. All procedures involving human participants/patients were approved by the Institutional Review Board of the New York State Psychiatric Institute, New York, NY, USA, in protocol number 6786. All participants provided informed written consent after an explanation of the study protocol and associated risks. Data on ethnicity were collected using self-report according to categories as required by the funding agency (National Institute of Mental Health).

### Assessment of childhood adversity and recent life stress

Childhood physical or sexual abuse was self-reported as present or absent during a semi-structured interview in 259 participants (93%) and was coded yes/no. Recent life stressors in the previous 2 years were recorded in 220 participants (79%) using the Recent Life Changes Questionnaire (RLCQ),^16^ which measures stressors in the previous four 6-month epochs on 76 items grouped in five domains: health; work; home/family; personal/social; and financial. Weights (in life change units, or LCU) for each item on the scale were as previously established.^17^

### Genotyping and methylation

Participants were genotyped at SNP rs6295 on the 5-HT_1A_ receptor gene (details are given in the Supplementary material, available at https://dx.doi.org/10.1192/bjp.2023.13). The Hardy–Weinberg equilibrium test in the control group was not significant (*P* = 0.084). DNA methylation at the −1019 CpG site was assayed except for GG genotypes, which are not methylated, and five other participants had missing values, at the −1007 CpG site in all participants and at the −681 CpG site in 279 (99.6%) of participants (see the Supplementary material for methodological details).

### PET measurement of 5-HT_1A_ binding potential

[^11^C]WAY100635 was administered as a bolus over 30 s for quantification of 5-HT_1A_ binding. Preparation of [^11^C]WAY100635 was as previously described.^18^ Radiotracer radioactivity in arterial plasma, parent fraction and free fraction in plasma (*f*_P_) were determined.^19^ Unmetabolised parent fraction levels were fit with a Hill function and then multiplied by the total radioactivity levels in plasma. The resulting metabolite-corrected arterial data were fitted using a straight line and the sum of three decreasing exponentials as the model before and after the curve peak respectively, to generate the final metabolite-corrected arterial input function (AIF). PET images were acquired with an ECAT EXACT HR+ scanner (Siemens/CTI, Knoxville, Tennessee). A T1-weighted magnetic resonance imaging (MRI) scan was acquired for each participant for co-registration with PET images. Thirteen brain regions of interest (ROIs) were chosen *a priori* based on areas of abundant binding: the raphe nuclei, anterior cingulate, cingulate, dorsal prefrontal cortex, hippocampus, insula, medial prefrontal cortex, parietal cortex, parahippocampal gyrus, occipital cortex, orbital cortex, temporal cortex and amygdala. Cerebellar white matter was used as a reference region.^13^ All ROIs except for the raphe nuclei were identified on each individual T1-weighted MRI using a previously described algorithm.^13^ Briefly, ROIs were drawn by trained technicians to approximate brain atlases and published reports and then refined using the segmented MRI. Owing to their small size, raphe nuclei were labelled using a standard space mask of the average location of the raphe nuclei in 52 healthy participants, created using [^11^C]WAY100635 voxel binding potential maps.^20^

#### Binding measure estimation

Arterial plasma radioactivity, metabolites and plasma free fraction (*f*_P_) were collected and assayed as previously described.^12^ Total volumes of distribution (*V*_T_) of [^11^C]WAY100635 were estimated for each ROI using the AIF and a two-tissue compartment constrained (2TCC) model. Briefly, each ROI's time activity curve was fitted with a 2TCC model in which the ratio of two of the model's free parameters (*K*_1_/*k*_2_, representing tracer non-displaceable volume of distribution) was constrained to equal that of the reference region. The outcome measure, binding potential BP_F_, was calculated as (*V*_T(ROI)_ – *V*_T(REF)_)/*f*_P_, where *V*_T(ROI)_ is the *V*_T_ in a specific ROI and *V*_T(REF)_ is the *V*_T_ in the reference region.

### Statistical method

All statistical analyses were performed in R version 4.09 run on MacOS.^21^ DNA methylation values and BP_F_ were log-transformed and outliers, defined as more than 1.5 interquartile range below the first quartile or above the third quartile, were censored to the nearest non-outlier value (less than 4% of values per variable). Genotype was coded as a three-level factor, although models with allele number were also explored. For models including methylation at the −1019 CpG site, GG genotypes were excluded, as that genotype cannot be methylated. Since childhood adversity was rare in the control group (reported by *n* = 5), all analyses involving childhood adversity were run only in the MDD group.

In preliminary analyses, logistic regression tested for association of childhood adversity history with genotype, age and gender. Recent stress in all participants was modelled in a multiple regression model as a function of genotype, diagnosis, age and gender.

The analysis was performed in three steps. First, associations between childhood adversity or recent stress and gene methylation level at the three sites were tested separately using linear regression, with methylation as dependent variable and stress/adversity, genotype, their interaction, age and gender as independent variables. Non-significant interactions were removed. Second, BP_F_ was modelled as a function of stress or adversity, genotype and their interaction, with age and gender as covariates. For recent stress, diagnostic group was included both as a main effect and through interaction with stress. Third, BP_F_ was modelled as a function of methylation, including group as main effect and in interactions. For BP_F_, first mixed-effects models were run including brain region as a main effect and through interaction with each predictor, and subject-specific random intercepts. Observations were weighted inversely proportionally to the squared standard errors of the BP_F_ estimates, calculated via bootstrapping, as described elsewhere.^22^ When interactions of predictors with brain region were significant, weighted least squares analyses were run at the region level. Analyses were not adjusted for multiple testing.

## Results

There were no significant differences between the MDD and control groups in age or gender (Table 1). Childhood physical or sexual abuse was reported by 6% of the control group, compared with 33% of the MDD group. Recent stress was slightly more severe in the MDD group compared with controls (Cohen's *d* = 0.46, *t* = 3.28, d.f. = 218, *P* = 0.0012), but in the MDD group did not correlate with depression severity on the Hamilton Rating Scale for Depression (Spearman *r*_s_ = 0.02, *P* = 0.8501) or with childhood adversity (*d* = 0.29, *t* = 1.56, d.f. = 132, *P* = 0.1219).

**Table 1:** Demographic and clinical characteristics of the sample (*n* = 280)

	*n*	Major depressive disorder group (*n* = 192)	Healthy volunteers (*n* = 88)	*P*
Age, years: mean (s.d.)	280	37 (12.5)	36 (13)	0.2800
Gender (male), *n* (%)	280	81 (42.4%)	40 (45.5%)	0.7453
Total education, years: mean (s.d.)	278	16 (3)	16 (2)	0.3810
Ethnicity	271			
American/Native		1 (0.5%)	0	**0.0005**^a^
Asian		4 (2.1%)	5 (5.9%)	
Black or African American		11 (5.9%)	10 (11.9%)	
White		166 (88.8%)	68 (81%)	
More than one ethnicity		5 (2.7%)	0	
Unknown		0	1 (1.2%)	
Childhood abuse, *n* (%)	258	56 (33%)	5 (5.7%)	**<0**.**0001**
17-item HRSD total, mean (s.d.)	280	18 (7)	2.5 (2)	**<0**.**0001**
Aggression history (BGLAH), mean (s.d.)	264	17 (4)	13 (3)	**<0**.**0001**
Barratt Impulsivity Scale, mean (s.d.)	230	50 (16)	36 (13)	**<0**.**0001**
Buss–Durkee Hostility Index, mean (s.d.)	237	30 (11)	18 (9)	**<0**.**0001**
Recent Life Changes Questionnaire, mean (s.d.)	220	501 (326)	364 (242)	**0**.**0012**

a. No significant pairwise differences were found between racial groups in *post hoc* comparisons with Holm's adjustment. Bold denotes significant group differences at the 5% level.

HRSD, Hamilton Rating Scale for Depression; BGLAH, Brown–Goodwin Lifetime Aggression History scale.

### Genotype, MDD diagnosis, childhood adversity and recent life stress

5-HT_1A_ receptor genotype distribution at SNP rs6295 did not differ between the MDD and control groups (χ^2^ = 2.55, d.f. = 2, *P* = 0.2788) or between those with and without reported childhood adversity in the MDD group (χ^2^ = 3.59, d.f. = 2, *P* = 0.1665). In a linear model adjusted for age and gender, recent stress was not associated with genotype in the full sample (*F* = 0.07; d.f. = 2,214; *P* = 0.9365) or in the MDD group (*F* = 0.15; d.f. = 2,135; *P* = 0.8590) and diagnosis-related differences remained significant after adjustment for genotype, age and gender (*F* = 11.5; d.f. = 1,213, *P* = 0.0008).

### Correlates of 5-HT_1A_ receptor gene methylation

Methylation levels at −1019 and −1007 CpG sites were modestly positively correlated with each other (Spearman *r*_s_ = 0.28, *P* < 0.0001), but neither correlated with methylation at the −681 CpG site (*r*_s_ = 0.10, *P* = 0.1319; *r*_s_ = 0.02, *P* = 0.7087).

Log-transformed methylation level measured at the −681 CpG site in a model with group and genotype as predictors, adjusting for age and gender, did not differ between groups (MDD versus control: *b* = −0.04, s.e. = 0.03, *t* = −1.16, d.f. = 272, *P* = 0.2479) and was unrelated to genotype (*F* = 0.76, d.f. = 2,272, *P* = 0.470). Methylation at −681 declined with age (*b* = −0.004, s.e. = 0.001, *t* = −3.35, d.f. = 272, *P* = 0.0009), but showed no gender differences (*b* = −0.01, s.e. = 0.03, *t* = −0.34, d.f. = 272, *P* = 0.7339). In the MDD group, −681 methylation was positively correlated with depression severity (Spearman *r*_s_ = 0.18, *P* = 0.0194) but not with childhood adversity (*b* = 0.02, s.e. = 0.04, *t* = 0.42, d.f. = 164, *P* = 0.6776). Across all participants, −681 methylation was positively correlated with recent stress in a model adjusted for group, genotype, age and gender (*b* = 0.0001, s.e. = 0.0001, *t* = 2.01, d.f. = 212, *P* = 0.0457). The strength of association did not differ by group (interaction *t* = −0.28, d.f. = 211, *P* = 0.7782).

**Fig. 1:**
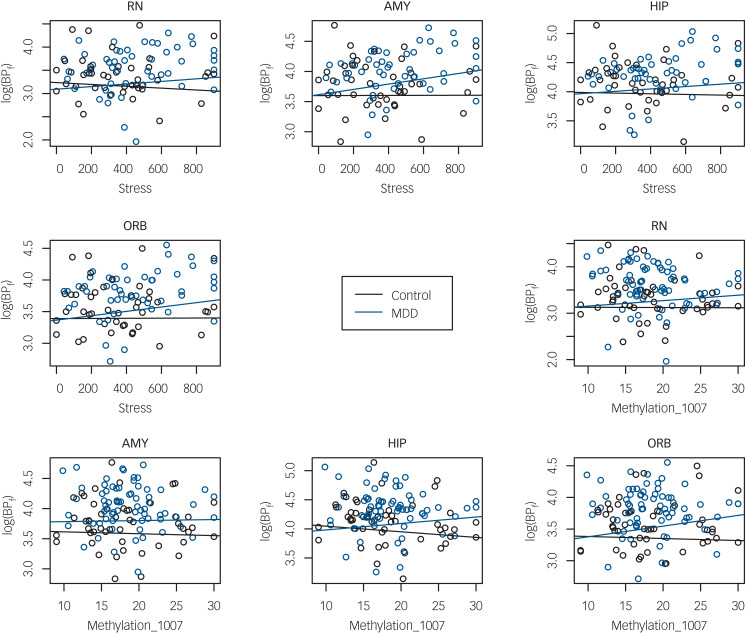
Serotonin 1A receptor binding potential (BP_f_) in four brain regions as a function of recent life stress and of methylation level at the 5-HT_1A_ promoter site −1007. RN, raphe nuclei; AMY, amygdala; HIP, hippocampus; ORB, orbital cortex; MDD, major depressive disorder.

DNA methylation level at the −1007 CpG site did not differ between diagnostic groups or between genotypes, neither was there a correlation with recent stress (Supplementary Table 1 and Fig. 1). In the MDD group, −1007 methylation was not associated with childhood adversity (*b* = 0.03, s.e. = 0.04, *t* = 0.71, d.f. = 164, *P* = 0.478), neither was it correlated with depression severity (Spearman *r*_s_ = −0.01, *P* = 0.8300).

DNA methylation at the −1019 CpG site (excluding the participants with GG genotype) did not differ by diagnostic group or correlate with recent stress (Supplementary Table 1 and Fig. 1). In the MDD group, adjusted for age and gender, methylation levels at the −1019 CpG was not associated with childhood adversity (*b* = 0.06, s.e. = 0.07, *t* = 0.94, d.f. = 134, *P* = 0.348), neither was there a correlation with depression severity (Spearman *r*_s_ = −0.02, *P* = 0.6400).

### 5-HT_1A_ binding potential: correlations with childhood adversity and recent stress, and group and genotype differences

In a model of the (log-transformed) [^11^C]WAY100635 BP_F_ in all regions, with genotype, diagnosis, age and gender as independent variables, we confirmed our previous finding of higher binding in the MDD group compared with controls across all brain regions (*b* = 0.23, s.e. = 0.07, *t* = 3.37, d.f. = 113, *P* = 0.0010) but did not find a main effect for genotype (*F* = 1.22; d.f. = 2,113; *P* = 0.3000). There was evidence of significant differences between brain regions in the diagnosis effect (*F* = 5.22, d.f. = 12,1356, *P* < 0.0001) and even in the genotype effect (*F* = 4.36, d.f. = 24,1356, *P* < 0.0001), although region-wise *post hoc* analyses (Supplementary Table 2) did not identify any significant genotype effects.

In the MDD group, there was evidence of differential association between adversity and BP_F_ by genotype and brain region (three-way interaction between region, childhood adversity and genotype *F* = 5.87; d.f. = 24,504; *P* < 0.0001). However, region-wise analyses did not find significant effect of childhood adversity or genotype effects on BP_F_ in any individual region, either as main effects or through interaction (Supplementary Fig. 3).

In the full sample, when testing association of BP_F_ with recent life stress, there was evidence of differential stress–BP_F_ association by genotype and brain region (three-way interaction *F* = 3.88; d.f. = 24,1056; *P* < 0.0001). Diagnosis also moderated the recent stress–BP_F_ association (three-way interaction *F* = 2.01, d.f. = 12,1080; *P* = 0.0210). To interpret these associations, separate analyses by diagnostic group and region were run to test the effect of stress on BP_F_ and a possible moderation by genotype. Region-wise analyses in the MDD group found that recent stress had positive association with BP_F_ in amygdala, orbital prefrontal cortex and insula (Table 2, Fig. 1, Supplementary Fig. 4) but no interactions were found between genotype and life stress, indicating that higher recent stress levels were associated with higher BP_F_ in these regions, regardless of genotype.

**Table 2:** Region-wise associations between recent life change (RLCQ) and receptor binding potential (BP_F_)^a^

Region	Major depressive disorder group			Healthy volunteers		
	Estimate	Standardised estimate	*P*	Estimate	Standardised estimate	*P*
Raphe nuclei	0.0005	0.35	0.09	−0.0002	−0.09	0.38
Amygdala	0.0004	0.38	0.02*	0.0001	0.04	0.77
Hippocampus	0.0003	0.28	0.06	−0.0001	−0.04	0.76
Parahippocampal gyrus	0.0001	0.11	0.49	−0.0001	−0.05	0.75
Temporal cortex	0.0003	0.25	0.11	0.0000	0.01	0.92
Anterior cingulate	0.0003	0.23	0.14	0.0000	0.01	0.96
Cingulate	0.0003	0.28	0.10	0.0000	−0.01	0.92
Dorsal prefrontal cortex	0.0003	0.25	0.11	0.0000	0.01	0.91
Medial prefrontal cortex	0.0003	0.25	0.11	0.0001	0.08	0.55
Orbital cortex	0.0004	0.34	0.04*	0.0000	0.02	0.84
Insula	0.0004	0.37	0.02*	0.0000	−0.02	0.89
Occipital cortex	0.0003	0.28	0.06	0.0000	0.02	0.85
Parietal cortex	0.0003	0.30	0.06	0.0000	<0.01	0.99

a. Separate analyses (adjusted for age and gender) are presented for participants with major depressive disorder and healthy volunteers. Outcome is log-transformed binding.

* Significant at the 5% level.

In contrast, in controls, no regions showed an association between recent stress and BP_F_; neither were there interaction effects with genotype (Table 2, Fig. 1, Supplementary Fig. 4).

### 5-HT_1A_ binding potential and DNA methylation

In a model of BP_F_ by methylation on the three sites and by diagnostic group, adjusted for genotype, age and gender, there was evidence of differential methylation effect by region and by group for two of the methylation sites (three-way interaction of region, diagnosis and methylation at the −1007 CpG site: *F* = 2.35; d.f. = 12,1044; *P* = 0.0057; and at the −681 CpG site: *F* = 6.17; d.f. = 12,1044; *P* < 0.0001). For the −1019 CpG site, the methylation–BP_F_ association differed by region but not by diagnostic group (methylation × region interaction: *F* = 8.54; d.f. = 12,1044; *P* = <0.0001). Region-wise analyses in each group run separately revealed that participants with MDD, but not controls, displayed a positive correlation of DNA methylation at the −1007 CpG site with BP_F_ in 7 of the 13 brain regions studied (raphe nuclei, hippocampus, cingulate, dorsolateral, medial prefrontal, orbital and parietal cortex); see Table 3 for numerical results, Fig. 1 for scatterplots of BP_F_ correlation with DNA methylation at the −1007 CpG site in four brain regions, and Supplementary Figs 5–7 for pairwise scatterplots of BP_F_ and DNA methylation level at each of the three sites in all brain regions.

**Table 3:** Effect sizes for region-wise associations between the three methylation sites and receptor binding potential (BP_F_)^a^

	Major depressive disorder group			Healthy volunteers		
Region	Methyl_681	Methyl_1007	Methyl_1019	Methyl_681	Methyl_1007	Methyl_1019
Raphe nuclei	0.32	0.49*	−0.21	−0.14	−0.03	0.03
Amygdala	0.24	0.22	<0.01	−0.17	−0.08	0.08
Hippocampus	0.30	0.33*	−0.02	−0.19	−0.19	0.06
Parahippocampal gyrus	0.16	0.29	0.13	−0.23	−0.20	0.12
Temporal cortex	0.25	0.32	0.06	−0.19	−0.12	0.10
Anterior cingulate	0.29	0.34	0.13	−0.19	−0.12	0.19
Cingulate	0.36	0.40*	0.09	−0.20	−0.17	0.18
Dorsal prefrontal cortex	0.24	0.35*	0.03	−0.18	−0.12	0.18
Medial prefrontal cortex	0.33	0.37*	0.03	−0.24	−0.16	0.15
Orbital cortex	0.39	0.47*	−0.07	−0.17	−0.15	0.13
Insula	0.42	0.32	0.08	−0.18	−0.13	0.11
Occipital cortex	0.29	0.29	0.07	−0.17	−0.11	0.15
Parietal cortex	0.39	0.39*	0.07	−0.20	−0.12	0.14

Methyl_, methylation level at the corresponding 5-HT_1A_ promoter site (−681, −1007, −1019).

a. Separate analyses (adjusted for age, gender and genotype; sample excludes GG genotypes) are presented for participants with major depressive disorder and healthy volunteers. Outcome is log-transformed binding. Effect sizes are standardised estimates of (log-transformed) binding difference for 1 standard deviation difference in methylation level.

* Significant at the 5% level.

Supplementary Fig. 8 summarises all model findings.

## Discussion

In this study, we report associations between recent stress and DNA methylation at the −681 CpG promoter site of the 5-HT_1A_ receptor gene. Additionally, in participants with MDD but not in controls, positive correlations were found between DNA methylation at the −1007 CpG 5-HT_1A_ receptor promoter site and higher 5-HT_1A_ receptor binding potential and also between recent stress and binding potential. However, since the findings occurred in disparate CpG sites, mediation could not be tested formally.

### Genotype, reported childhood adversity, recent stress and MDD

Our finding that a history of childhood adversity was essentially restricted to the MDD group is consistent with previous reports.^23^,^24^ In addition, our findings of no association between 5-HT_1A_ receptor promoter genotype and history of childhood adversity in the MDD group and no association with recent stress in either the full sample or the MDD group alone are consistent with a previous study in healthy volunteers.^25^ Others report an association of the G-allele at the C(−1019)G site with greater stress reactivity, depression and suicide in adulthood.^26–28^

### 5-HT_1A_ receptor DNA methylation, reported childhood adversity, recent life stress and MDD

Childhood adversity was not related to DNA methylation levels at the three promoter sites (−1019, −1007, −681) in peripheral blood monocytes in either the MDD group or controls; however, we cannot rule out DNA methylation effects of childhood adversity on the brain.

Recent stress was positively correlated with DNA methylation at the −681 CpG site, and this was not altered after controlling for group, genotype, age and gender. In contrast, DNA methylation at the −1019 and −1007 sites did not correlate with the −681 CpG or recent life stress. Similarly to our findings in humans, in stress-sensitive adult BALB/c mice, chronic mild unpredictable stress increased DNA methylation at the −681 CpG site.^10^

### 5-HT_1A_ receptor binding potential, MDD, DNA methylation and stress

We found that recent stress had a positive association with 5-HT_1A_ receptor binding potential in the amygdala, orbital cortex and insula in the MDD group, consistent with the report that chronic adult stress increased 5-HT_1A_ receptor RNA and receptor levels in both medial prefrontal cortex and midbrain in stress-sensitive BALB/c mice.^10^ In the same study, increased 5-HT_1A_ receptor binding was accompanied by an increase in DNA methylation at the −681 CpG site, which is consistent with our finding of a positive correlation of DNA methylation at the −681 CpG site with recent stress, although our findings are for peripheral methylation. However, in our study, only methylation at the −1007 site was positively correlated with PET binding in *post hoc* testing. The effects of peripheral DNA methylation at the −681 site appeared to be more variable among individual brain regions because of a complex three-way group × region × stressor interaction effect. This complexity meant that we could not posit a simple mediation model in which the effect of recent stress on 5-HT_1A_ receptor binding is mediated through elevated DNA methylation levels at the −681 and/or −1007 CpG sites.

### Limitations and strengths

A limitation of this study is that it was not possible to measure DNA methylation in brain *in vivo,* so we used peripheral blood monocyte DNA to assay methylation. Additionally, even though this is one of the largest PET studies of MDD, the numbers are still limited for an epigenetic study and we therefore did not adjust for multiple testing. We did not include participants with other Axis I disorders and did not adjust for comorbid axis II disorders such as borderline personality disorder. Our stress measurement method could not determine whether stress might be caused by the depressed state itself. Last, childhood adversity in this sample was almost exclusively reported in the MDD group and therefore its effect in controls could not be reported. Strengths of the study include the large sample of participants undergoing PET scans and that the MDD group were off antidepressant medication at the time of study.

## Data Availability

The de-identified data that support the findings of this study are available on reasonable request from the corresponding author (H.G.). The data are not publicly available because the participants in the protocol did not agree to share their data publicly.

## Supplementary material

For supplementary material accompanying this paper visit https://doi.org/10.1192/bjp.2023.13. click here to view supplementary material
