# Supplementary material for: Brain serotonin 1A receptor binding: relationship to peripheral blood DNA methylation, recent life stress and childhood adversity in unmedicated major depression
Source: Br J Psychiatry. 2023 Sep;223(3):415–21. doi: 10.1192/bjp.2023.13 (PMC10514224; doi:10.1192/bjp.2023.13)

Supplement

Methylation analysis of 5-HT$_{1A}$ receptor promoter region by Bisulfite Pyrosequencing

Briefly, the percentage of CpG methylation at selected sites were quantified by bisulfite conversion of genomic DNA followed by PCR amplification and pyrosequencing[1]. 500ng of genomic DNA from the PBMCs fraction of blood samples were bisulfite-treated (EpiTect Bisulfite kit, Qiagen) and stored at -20^{0}C until further analysis. The bisulfite converted DNA was amplified by PCR thermocycler using the PyroMark PCR kit (Qiagen) according to manufacturer’s instructions. For the -1019CpG and -1007CpG sites, PCR primer set was designed as (Forward primer: 5’-TTTGTAGTTTAGGTTGGAGTGTAATG-3’; reverse primer: 5’-CCTAAATCAATCTCCCAATTATTACTAA-3’[biotinylated] and Sequence primer: 5’-CCTAAATCAATCTCCCAATTATTACTAA-3’). For the -681CpG site, PCR and sequencing primers are as follow: Forward primer: 5’-TGATAGGTAGGGATTTGGTGTGTGT-3’, reverse primer: 5’-CCCTCCCCTCCTAATACTT-3’and sequence primer: 5’-CCAACCCCCAAAAATACC-3’. Selection of the primers were generated with the PyroMark Assay Design software (Version 2.0; Qiagen) and PCR products were first tested with PCR protocol to determine the suitability of the PCR products for the sequence. Pyrosequence is performed using a PyroMark Q96 MD system (Qiagen). Percent methylation at each CpG site was quantified using the PyroMark CpG software (Version 1.0.10, Qiagen).

Reference:

1. Dupont JM, Tost J, Jammes H, Gut IG. De novo quantitative bisulfite sequencing using the pyrosequencing technology. Anal Biochem. 2004 Oct 1;333(1):119-27. doi: 10.1016/j.ab.2004.05.007.PMID: 15351288

Primer sets for 5-HT$_{1A}$ promoter region: -681, -1007 & -1019 CpG sites, rs6295 (-C1019G) SNP

↪	MHTR1A_F2	[Btn] 5’-TGATAGGTAGGGATTTGGTGTGTGT
↼	MHTR1A_R2	5’-CCCTCCCCTCCTAATACTT-3’
←	MHTR1A_S2	5’-CCAACCCCCAAAAATACC-3’

5-HT$_{1A}$ promoter region -681 CpG (Pyrosequencing, Methylation)

⇀	MHTR1A_F1	5'-AGTAAGGTTGGATTGTTAGATGA-3'
↽	MHTR1A_R1	5'-CCTAAATCAATCTCCCAATTATTACTAA-3'[Btn]
→	MHTR1A_S1	5'-GAGAAAGGAGGTAGTTTTTTAA-3'

5-HT_{1A} promoter region -1019, -1007 CpG (Pyrosequencing, Methylation)

Mann-42	HTR1A_F1	5'-CTGAGGGAGTAAGGCTGGAC-3'
Mann-43	HTR1A_R1	5'-GAAGACCGAGTGTGTCTTCG-3' for the C allele
Mann-44	HTR1A_R2	5'-GAAGACCGAGTGTGTCTTCC-3' for the G allele

5-HT_{1A} promoter region **rs6295 (-C1019G)** SNP (Genotyping)

Suppl. Table S1. DNA methylation on three 5HT1A promoter sites: associations with recent life stress, diagnostic group and genotype (adjusted for age and sex). Methylation was log-transformed, model for site -1019 excludes those with GG genotype on rs6295.

Predictors	Estimate	Std. Error	t value	p-value
**Site -681**				
MDD	-0.04	0.03	-1.07	0.2876
Recent Stress	0.00	0.00	2.01	0.0457
GenotypeCG	-0.02	0.04	-0.45	0.6554
GenotypeGG	0.00	0.05	0.06	0.9485
Age	0.00	0.00	-2.34	0.0203
Male	-0.04	0.03	-1.27	0.2051
**Site -1017**				
MDD	0.04	0.04	0.95	0.3456
Recent Stress	0.00	0.00	0.86	0.3883
GenotypeCG	-0.02	0.04	-0.59	0.5564
GenotypeGG	-0.02	0.06	-0.35	0.7244
Age	0.00	0.00	0.44	0.6571
Male	0.02	0.04	0.64	0.5202
**Site -1019**				
MDD	-0.05	0.06	-0.87	0.3839
Recent Stress	0.00	0.00	0.35	0.7278
GenotypeCG	0.15	0.06	2.59	0.0105
Age	0.00	0.00	0.69	0.4932
Male	-0.01	0.06	-0.25	0.8010

Suppl. Table S2. Estimates for region-wise associations of diagnostic group and genotype with binding (adjusted for age and sex).

	MDD (yes/no)		Genotype CG		Genotype GG	
**Region**	**Estimate**	**p-value**	**Estimate**	**p-value**	**Estimate**	**p-value**
**RN**	0.14	0.08	0.09	0.26	-0.28	0.03 *
**AMY**	0.17	0.01 *	0.05	0.44	0.01	0.91
**HIP**	0.07	0.26	0.03	0.71	0.01	0.92
**PIP**	0.17	0.01 **	0.03	0.68	<0.01	0.98
**TEM**	0.15	0.02 *	<0.01	0.94	0.05	0.67
**ACN**	0.14	0.02 *	-0.01	0.92	0.08	0.47

	MDD (yes/no)		Genotype CG		Genotype GG	
**Region**	**Estimate**	**p-value**	**Estimate**	**p-value**	**Estimate**	**p-value**
**CIN**	0.14	0.04 *	-0.06	0.41	0.02	0.88
**DOR**	0.16	0.01 **	<0.01	0.99	0.07	0.55
**MED**	0.15	0.02 *	-0.01	0.91	0.11	0.33
**ORB**	0.13	0.04 *	-0.02	0.81	0.04	0.74
**INS**	0.07	0.27	-0.01	0.85	0.06	0.60
**OCC**	0.15	0.02 *	-0.01	0.90	0.11	0.34
**PAR**	0.14	0.03 *	-0.02	0.74	0.04	0.71

NOTE: Outcome is log-transformed binding.

Significant at the * 5% level; ** 1% level.

Supplementary Figure S1. **Methylation levels on site 681,1007 and 1019, by subjects' scores on the Recent Life Changes Questionnaire.**

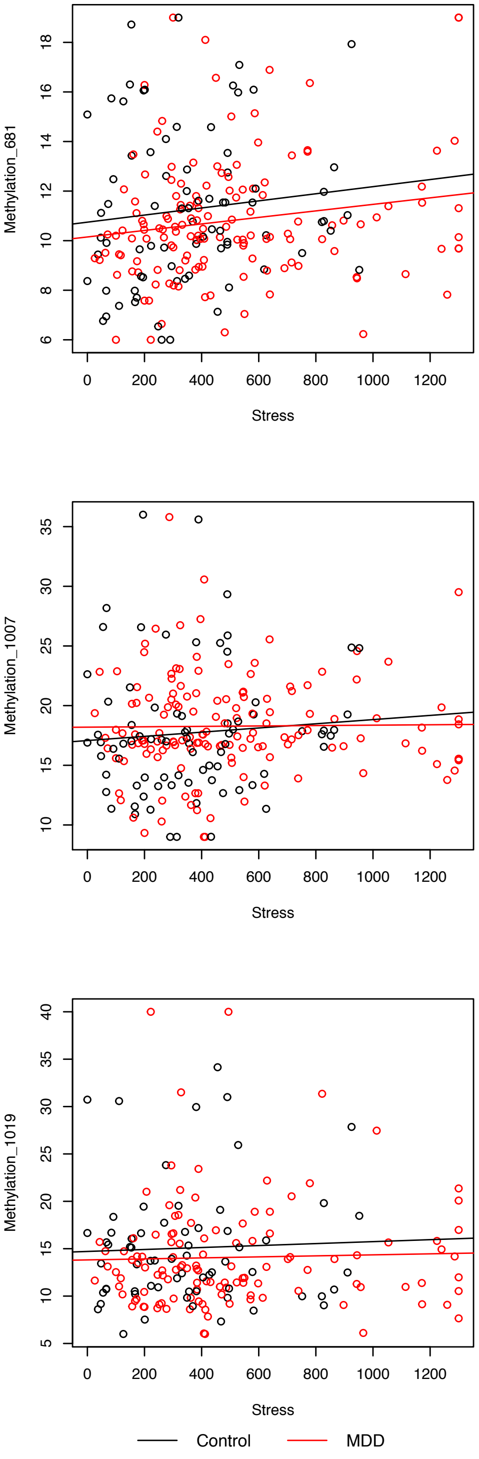

Supplementary Figure S2. Least-square estimates of region-wise WAY binding by diagnostic group, illustrating higher binding in MDD subjects.

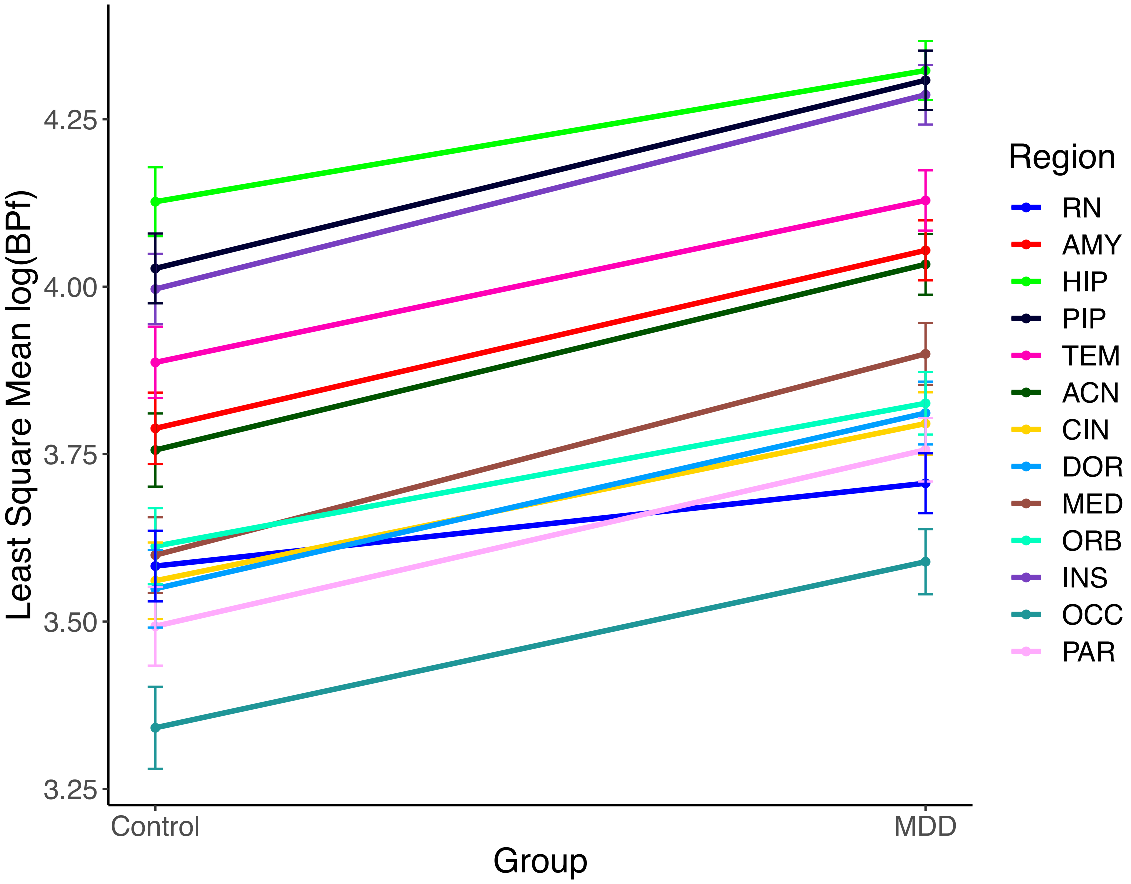

Supplementary Figure S3. Least-square estimates of region-wise WAY binding by childhood abuse, in MDD subjects.

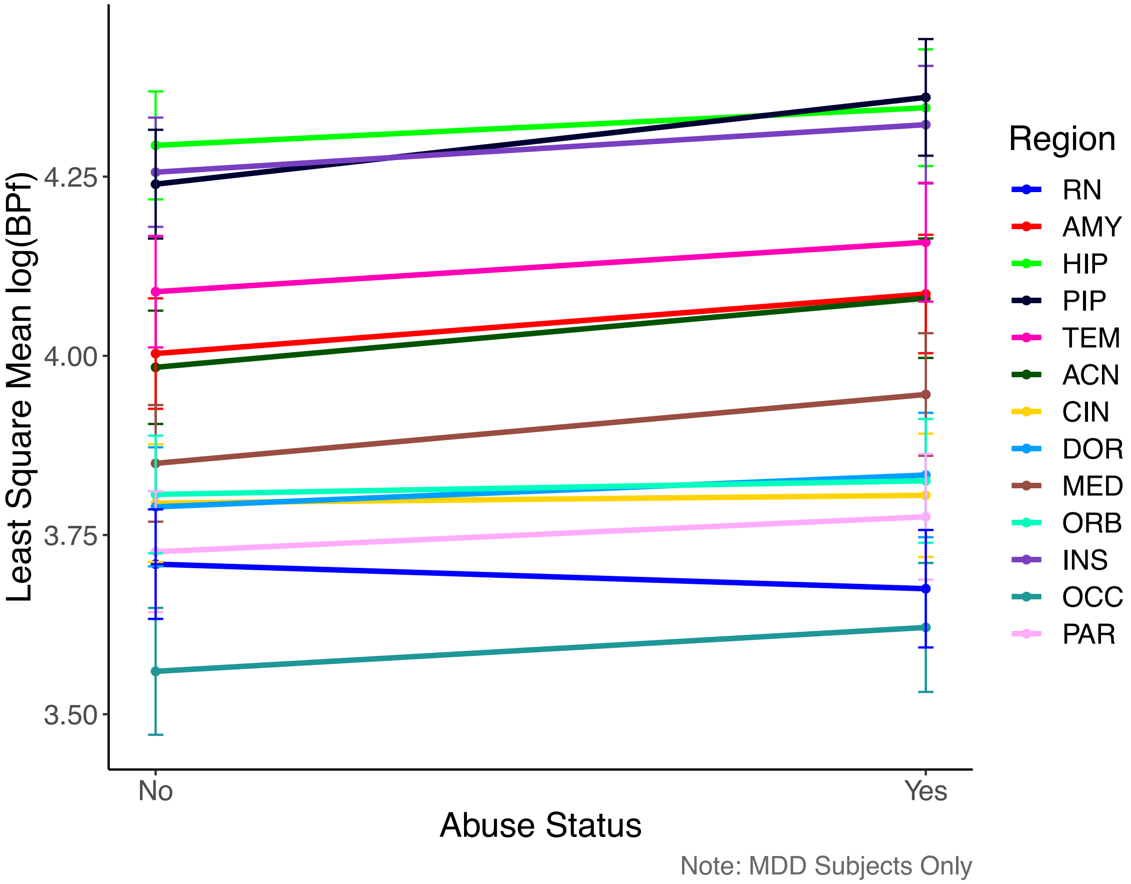

Supplementary Figure S4. WAY binding levels, as a function of recent life stress, in all 13 brain regions.

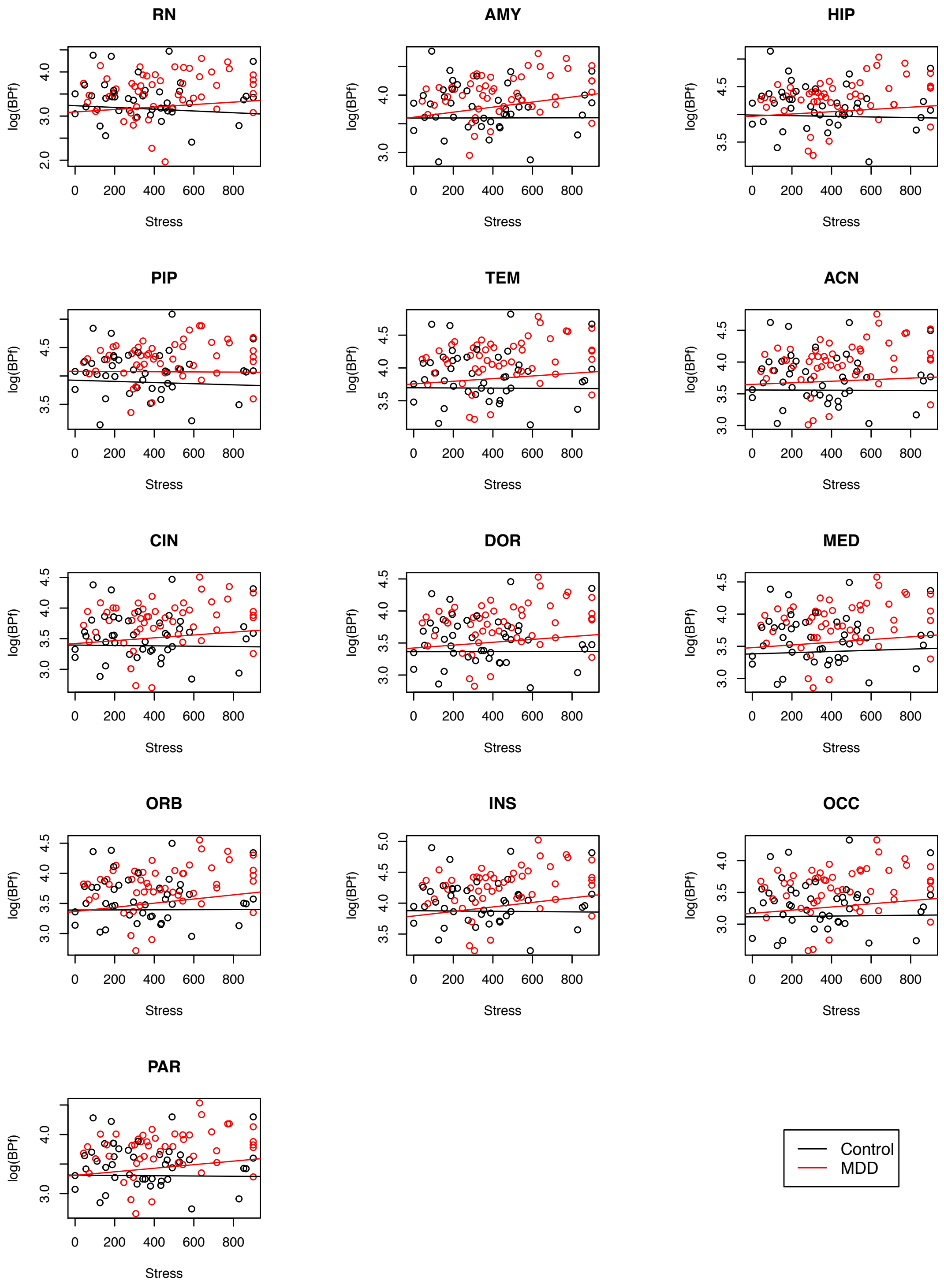

Supplementary Figure S5. WAY binding levels, as a function of methylation level on site 1007, in all 13 brain regions. Only subjects without the GG genotype were included.

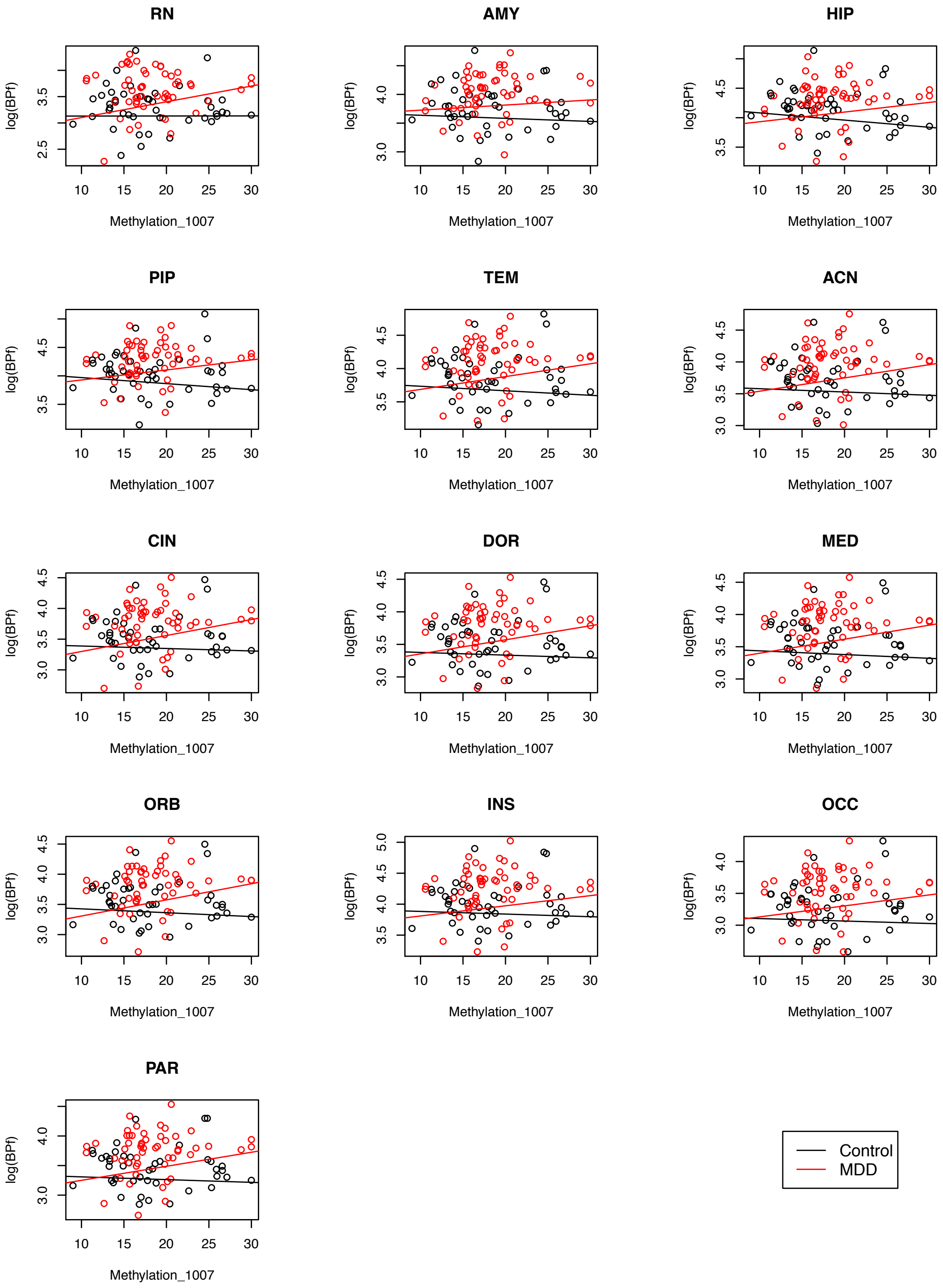

Supplementary Figure S6. WAY binding levels, as a function of methylation level on site 681, in all 13 brain regions. Only subjects without the GG genotype were included.

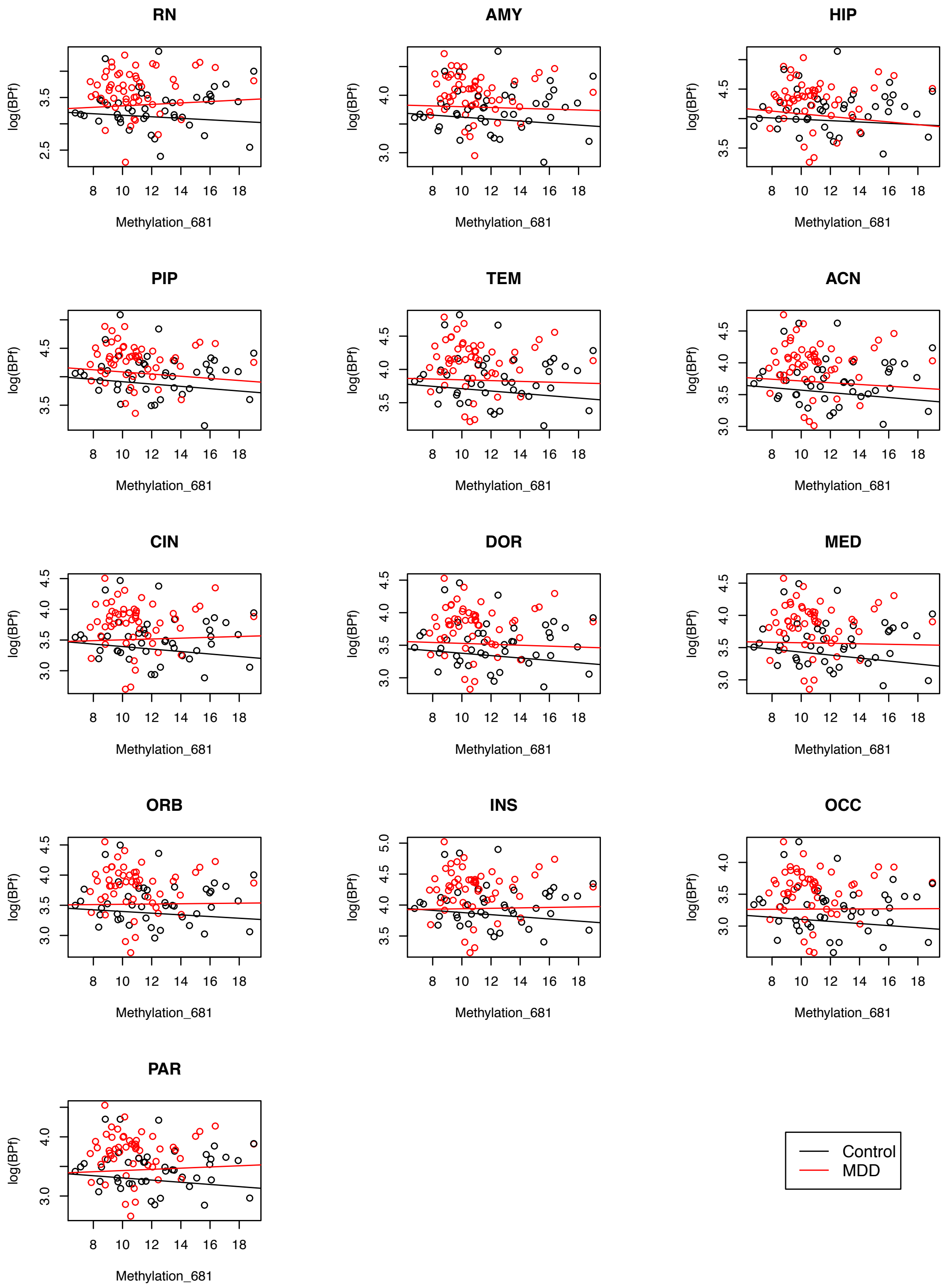

Supplementary Figure S7. WAY binding levels, as a function of methylation level on site 1019, in all 13 brain regions. Only subjects without the GG genotype were included.

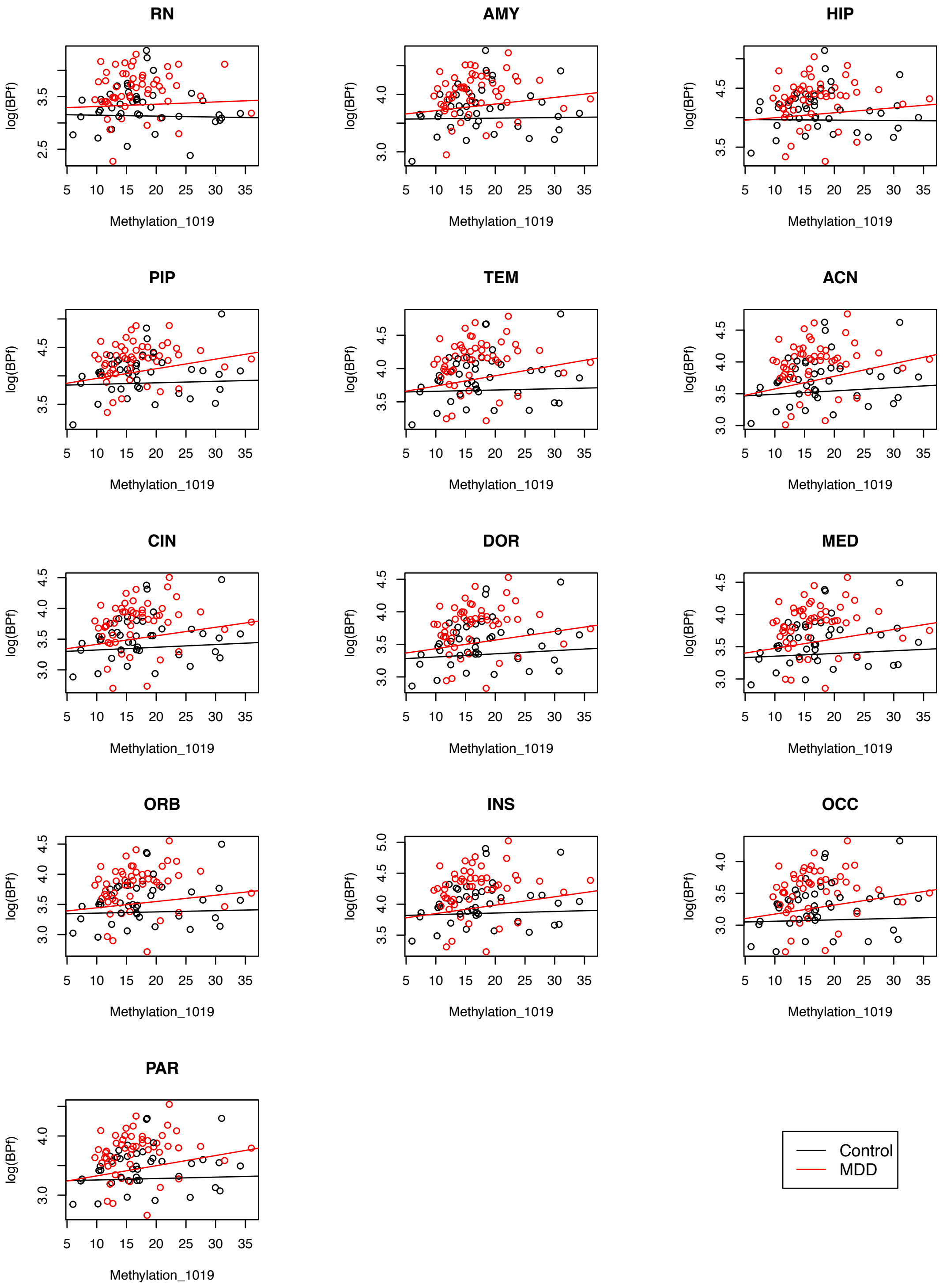

Figure S8. Model of the associations found significant in the study. Solid lines denote significant associations, dashed line denotes an association that was part of a significant interaction in the all-regions analysis, but not in region-wise analyses.

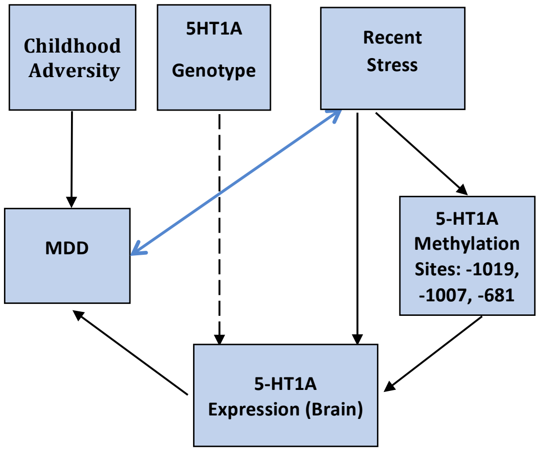

Supplement: Supplementary file 1 [file S0007125023000132sup001.pdf]
